# ^212^Pb-Labeled Antibody 225.28 Targeted to Chondroitin Sulfate Proteoglycan 4 for Triple-Negative Breast Cancer Therapy in Mouse Models

**DOI:** 10.3390/ijms19040925

**Published:** 2018-03-21

**Authors:** Benjamin B. Kasten, Patsy G. Oliver, Harrison Kim, Jinda Fan, Soldano Ferrone, Kurt R. Zinn, Donald J. Buchsbaum

**Affiliations:** 1 Department of Radiology, University of Alabama at Birmingham, Birmingham, AL 35294, USA; hyunkikim@uabmc.edu (H.K.); jindafan@uabmc.edu (J.F.); 2 Department of Radiation Oncology, University of Alabama at Birmingham, Birmingham, AL 35294, USA; poliver@uab.edu (P.G.O.); djb@uab.edu (D.J.B.); 3 Department of Surgery, Massachusetts General Hospital, Harvard Medical School, Boston, MA 02114, USA; SFERRONE@mgh.harvard.edu; 4 Institute for Quantitative Health Science and Engineering, Department of Radiology, Michigan State University, East Lansing, MI 48824, USA; zinnkurt@egr.msu.edu

**Keywords:** radioimmunotherapy, triple-negative breast cancer, ^212^Pb, CSPG4

## Abstract

Triple-negative breast cancer (TNBC) is an aggressive subtype of breast cancer with a poor prognosis. There is a clinical need for effective, targeted therapy strategies that destroy both differentiated TNBC cells and TNBC cancer initiating cells (CICs), as the latter are implicated in the metastasis and recurrence of TNBC. Chondroitin sulfate proteoglycan 4 (CSPG4) is overexpressed on differentiated tumor cells and CICs obtained from TNBC patient specimens, suggesting that CSPG4 may be a clinically relevant target for the imaging and therapy of TNBC. The purpose of this study was to determine whether α-particle radioimmunotherapy (RIT) targeting TNBC cells using the CSPG4-specific monoclonal antibody (mAb) 225.28 as a carrier was effective at eliminating TNBC tumors in preclinical models. To this end, mAb 225.28 labeled with ^212^Pb (^212^Pb-225.28) as a source of α-particles for RIT was used for in vitro Scatchard assays and clonogenic survival assays with human TNBC cells (SUM159 and 2LMP) grown as adherent cells or non-adherent CIC-enriched mammospheres. Immune-deficient mice bearing orthotopic SUM159 or 2LMP xenografts were injected *i.v.* with the targeted (225.28) or irrelevant isotype-matched control (F3-C25) mAbs, labeled with ^99m^Tc, ^125^I, or ^212^Pb for in vivo imaging, biodistribution, or tumor growth inhibition studies. ^212^Pb-225.28 bound to adherent SUM159 and 2LMP cells and to CICs from SUM159 and 2LMP mammospheres with a mean affinity of 0.5 nM. Nearly ten times more binding sites per cell were present on SUM159 cells and CICs compared with 2LMP cells. ^212^Pb-225.28 was six to seven times more effective than ^212^Pb-F3-C25 at inhibiting SUM159 cell and CIC clonogenic survival (*p* < 0.05). Radiolabeled mAb 225.28 showed significantly higher uptake than radiolabeled mAb F3-C25 in SUM159 and 2LMP xenografts (*p* < 0.05), and the uptake of ^212^Pb-225.28 in TNBC xenografts was correlated with target epitope expression. ^212^Pb-225.28 caused dose-dependent growth inhibition of SUM159 xenografts; 0.30 MBq ^212^Pb-225.28 was significantly more effective than 0.33 MBq ^212^Pb-F3-C25 at inhibiting tumor growth (*p* < 0.01). These results suggest that CSPG4-specific ^212^Pb-225.28 is a useful reagent for RIT of CSPG4-expressing tumors, including metastatic TNBC.

## 1. Introduction

Triple-negative breast cancer (TNBC) is an aggressive subtype of breast cancer characterized by a lack of progesterone receptor and estrogen receptor alpha expression and by no or low amplification of human epidermal growth factor receptor 2 (HER-2) [1]. There are currently no approved targeted therapies for TNBC, which contributes to the poor prognosis for the nearly 20% of patients with this subtype of breast cancer [1,2]. Cancer initiating cells (CICs), also referred to as cancer stem cells, are recognized as a significant problem in effectively managing breast cancer. CICs are known for their resistance to chemotherapy, ability to form non-adherent mammospheres in vitro, and high tumorigenicity in vivo [3,4,5,6,7]. Standard chemotherapies (e.g., taxanes) have been shown to enrich the population of CICs in TNBC [7,8,9]. These surviving CICs are implicated in generating chemotherapy-resistant, recurrent, and metastatic disease [1,3,10]. Therefore, there is a clinical need to develop novel, targeted approaches that kill both differentiated TNBC cells and CICs, particularly in recurrent and metastatic lesions, to improve the outcomes for patients with TNBC.

Therapeutic strategies utilizing high linear energy transfer (LET) α-particles (100 keV/μm) are advantageous for killing individual cells and microscopic clusters of cells while in circulation (e.g., early metastases) regardless of the cells’ sensitivity to chemotherapy or to alternative low LET radiation (external beam or β-particles, 0.1–1 keV/μm) [11,12,13,14]. The majority of TNBC tumors (>70%) contain *BRCA-1* mutations that impair their ability, compared to normal cells, to repair double strand DNA breaks caused by α-particles, thus potentially augmenting the therapeutic window for α-particle therapy of TNBC [15]. Results from preclinical and clinical studies using the α-particle emitter ^223^Ra to treat bone metastases of breast cancer support the utility of α-particle radionuclides against refractory breast cancer [16,17]. The success of systemically administered ^223^Ra against bone metastases is due to localization of the radiometal to areas of bone restructuring and to the short range (<100 μm) of α-particles, which minimizes their penetration into adjacent healthy tissues. Due to the inability of ^223^Ra to accumulate in primary tumors or non-skeletal metastases, alternative radionuclides and targeting strategies are required to be effective against primary TNBC and metastases to other organs. Many monoclonal antibodies (mAbs) that specifically bind to antigens overexpressed on malignant cells have been used as carriers for α-particle emitting nuclides in targeted radioimmunotherapy (RIT) studies against preclinical in vitro and in vivo models of breast cancer [11,12,14,18,19,20,21,22,23,24,25,26,27]. However, no existing mAbs or radioimmunoconjugates (RICs) that simultaneously target differentiated, bulk tumor cells and CICs are approved for TNBC or other types of breast cancer.

Chondroitin sulfate proteoglycan 4 (CSPG4), also referred to as high-molecular-weight melanoma-associated antigen, has gained recognition as a biomarker for imaging and therapeutic applications in several types of malignancies, including TNBC [4,5,28,29]. High levels of CSPG4 are expressed on invasive, chemotherapy-resistant, differentiated malignant cells and CICs, while the expression on indolent cancer cells and normal tissues is low [4]. Previous studies using CSPG4-specific mAbs have shown that CSPG4 is expressed on nearly 73% of primary TNBC patient specimens, malignant cells in pleural effusions from patients with TNBC, human TNBC cells and CICs cultured in vitro, and TNBC xenograft tumors grown in immunodeficient mice [5,28,30]. Employing CSPG4-specific mAbs as carriers for diagnostic or cytotoxic agents, such as radionuclides, represents an attractive strategy to specifically target aggressive TNBC differentiated cells, CICs, and metastatic cells for imaging or therapeutic applications. mAb 225.28 binds to a specific epitope of CSPG4 that is highly expressed on the surface of human TNBC cells and CICs but has limited expression in normal tissues [5,28,30]. These factors suggest that labeling mAb 225.28 with ^212^Pb (*t*_1/2_ = 10.64 h), which decays to the α-particle emitter ^212^Bi (*t*_1/2_ = 60.5 min), would generate a CSPG4-targeted α-particle RIC that could effectively kill TNBC cells and CICs, which are implicated in the poor outcomes associated with TNBC. The purpose of the studies described here was to test the specific binding, localization, and therapeutic efficacy of CSPG4-targeted RICs of mAb 225.28 in preclinical in vitro and in vivo mouse models of human TNBC, in order to determine if this is a valid approach for future clinical development.

## 2. Results

### 2.1. In Vitro Differential Expression of CSPG4 on Human TNBC Cell Lines

^212^Pb-225.28 bound to adherent SUM159 and 2LMP cells and to CICs dissociated from SUM159 or 2LMP mammospheres with an affinity of 0.5 ± 0.1 nM (mean ± SEM for all adherent cells and CICs) and had a specific binding level above 83% (Table 1, Supplementary Figure S1). Scatchard analyses indicated there were nearly ten times more binding sites per cell on the SUM159 cells compared to the 2LMP cells. This result indicates that the expression of the CSPG4 epitope defined by mAb 225.28 was higher on SUM159 cells than on 2LMP cells. For both cell lines, CICs had 1.7–2.3 times more binding sites per cell than adherent cells. Due to the high number of binding sites on the SUM159 cells, further in vitro studies with this cell line were performed.

### 2.2. ^212^Pb-225.28 Reduces the In Vitro Proliferation of Human TNBC Differentiated Cells and CICs

^212^Pb-225.28 significantly inhibited the in vitro clonogenic survival of adherent SUM159 cells and CICs from SUM159 mammospheres compared to the irrelevant isotype-matched control, ^212^Pb-F3-C25 (Table 2; *p* < 0.05, Student’s *t-*test). The concentration of ^212^Pb-225.28 that reduced adherent cell and CIC colony formation by 50% (IC_50_) compared to non-treated controls was 6.6 to 7.2 fold lower than the concentration of ^212^Pb-F3-C25 that produced the same effect. SUM159 CICs were 1.8 times more sensitive than adherent cells to treatment with ^212^Pb-225.28.

### 2.3. Tumor Size Influences the Uptake of Radiolabeled mAb 225.28 in Human TNBC Xenografts

**Figure 1:**
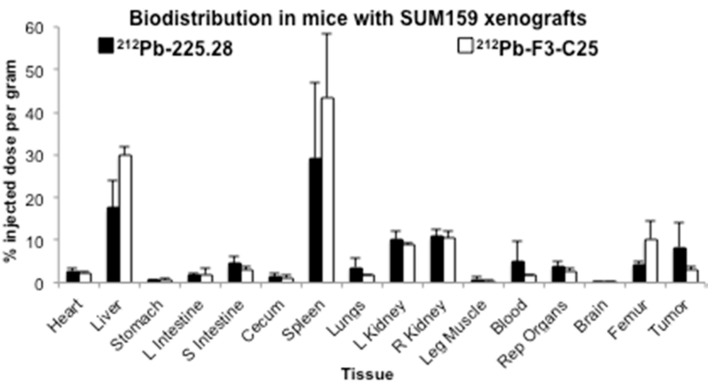
Biodistribution results at 24 h after *i.v.* injection of 0.78 MBq ^212^Pb-225.28 or ^212^Pb-F3-C25 in groups of athymic nude mice (*n* = 4/group) bearing small SUM159 xenografts implanted in the mammary fat pad. Data are presented as mean % ID/g ± standard deviation, and the difference in the tumor uptake between the two groups was analyzed by Student’s *t*-test (not significant).

Biodistribution study 1 with ^212^Pb-225.28 or ^212^Pb-F3-C25 was performed in mice bearing small (mean 67 mm^3^) SUM159 xenografts in the mammary fat pad, when tumors had not yet reached the initial log-phase of growth. At 24 h after injection of the RICs, tumors from mice dosed with ^212^Pb-225.28 showed higher uptake of the injected ^212^Pb activity (8.3 ± 5.7% ID/g) compared to tumors from mice dosed with ^212^Pb-F3-C25 (3.0 ± 0.7% ID/g) (Figure 1, Supplementary Table S1), although the difference was not significant (*p* = 0.116, Student’s *t-*test). Compared to other normal organs, the spleen, liver, and kidneys of mice from both ^212^Pb-225.28 and ^212^Pb-F3-C25 dose groups showed high uptake of ^212^Pb. While most normal tissues from both groups of mice showed comparable uptake of the injected ^212^Pb dose at 24 h after injection, higher ^212^Pb activity was observed in the spleens, livers, and femurs from mice dosed with ^212^Pb-F3-C25 compared to mice dosed with ^212^Pb-225.28 (Figure 1, Supplementary Table S1).

**Figure 2:**
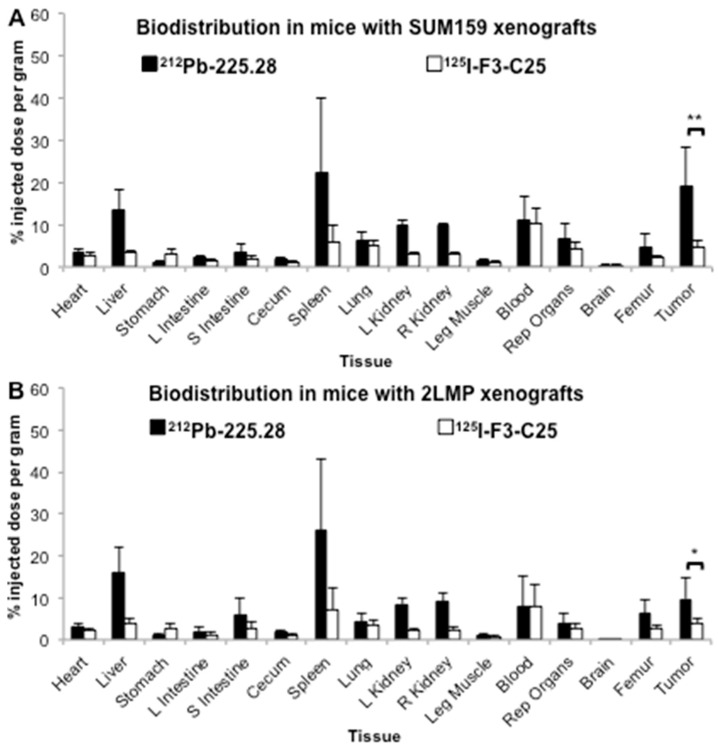
Biodistribution results in athymic nude mice bearing human TNBC xenograft tumors. Groups of mice (*n* = 5/group) bearing large SUM159 xenografts (**A**) or 2LMP xenografts (**B**) were injected *i.v.* with a solution containing 0.68 MBq ^212^Pb-225.28 and 16.3 kBq ^125^I-F3-C25. Mice were euthanized at 24 h post injection, when selected tissues were removed, weighed, and counted with a gamma counter to determine the % ID/g of tissue for each radionuclide. ^125^I was counted after ^212^Pb had fully decayed. Data are presented as means ± standard deviations, and the difference in the uptake between ^212^Pb and ^125^I in each group of tumors was analyzed by Student’s *t*-tests. * *p* < 0.05; ** *p* < 0.01.

Biodistribution study 2 utilized a co-injection of ^212^Pb-225.28 and ^125^I-F3-C25 in mice bearing large (mean ~120 mm^3^) SUM159 or 2LMP xenografts in the mammary fat pad when tumors were entering the initial log-phase of growth. This experiment was designed to test (a) if the specific uptake of activity from ^212^Pb-225.28 in SUM159 tumors is higher in larger tumors compared to the small tumors in study 1; and (b) if the specific uptake of ^212^Pb-225.28 in TNBC xenografts correlates with the relative epitope expression observed on the cell lines in vitro (Table 1: high in SUM159 model, low in 2LMP model). Utilizing different radionuclides on the CSPG4-targeted and isotype control mAbs enabled the uptake of the two RICs to be directly compared in the same tissues from each mouse. Biodistribution results at 24 h after injection of the RICs in these studies are provided in Supplementary Tables S2 and S3. As shown in Figure 2, both types of TNBC xenografts showed significantly greater uptake of the injected activity from ^212^Pb-225.28 compared to the activity from the control, ^125^I-F3-C25 (SUM159, 19.0 ± 9.2% vs. 4.7 ± 1.7% ID/g, respectively, *p* < 0.01; 2LMP, 9.7 ± 4.9% vs. 4.0 ± 1.1% ID/g, respectively, *p* < 0.05), at 24 h after dosing. The uptake of ^212^Pb activity in the large SUM159 xenografts (Figure 2A) was nearly twice as high as in the large 2LMP xenografts (Figure 2B) and was higher than that observed in the small SUM159 xenografts in biodistribution study 1 with ^212^Pb-225.28 (Figure 1). Comparable amounts of the injected ^212^Pb and ^125^I activities were present in the blood at the 24 h time point. Most normal organs, particularly those in the reticuloendothelial system (RES), showed higher uptake of the injected activity from ^212^Pb-225.28 compared to activity from ^125^I-F3-C25 in these sets of mice. Conversely, higher uptake of the injected ^125^I activity compared to ^212^Pb was observed in the stomach (Supplementary Tables S2 and S3), which suggests partial dehalogenation from ^125^I-F3-C25 in vivo or during storage between the labeling procedure and the biodistribution study (15-day lapse).

**Figure 3:**
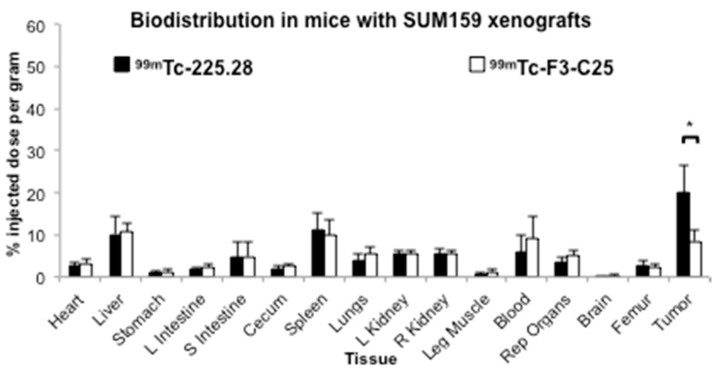
Biodistribution results at 24 h after injection of ^99m^Tc-225.28 or ^99m^Tc-F3-C25 in the groups of athymic nude mice (*n* = 3–4/group) bearing large SUM159 xenografts implanted in the mammary fat pad. Mice were euthanized at 24 h post injection, when selected tissues were removed, weighed, and counted in a gamma counter to determine the % ID/g of tissue. Data are presented as means ± standard deviations, and the difference in the tumor uptake between the two groups was analyzed by Student’s *t*-tests. * *p* < 0.05.

The imaging and biodistribution study 3 with ^99m^Tc-225.28 or ^99m^Tc-F3-C25 was performed in groups of mice bearing medium–large (mean 94 mm^3^) SUM159 xenografts in the mammary fat pad (Figure 3, Supplementary Figure S2 and Table S4), when tumors were entering the log-phase of growth. This study was not able to be performed during the lifetime of the ^224^Ra/^212^Pb generators; thus, the readily available radionuclide ^99m^Tc was used to label the targeted and control RICs. This study also investigated whether the mild HYNIC conjugation and ^99m^Tc labeling conditions would result in less RES uptake of activity compared to the ^212^Pb-RICs in studies 1 and 2. In vivo planar gamma camera imaging at 23 h after dosing mice with ^99m^Tc-225.28 showed marked uptake of radioactivity in the tumors, with a low signal in normal tissues in the abdominal region (Supplementary Figure S2A). The tumors in mice dosed with ^99m^Tc-F3-C25 showed no appreciable accumulation of activity compared to the surrounding tissues (Supplementary Figure S2B). Subsequent ex vivo biodistribution analyses were consistent with the imaging analyses. The relative uptake of activity at 24 h in the xenograft tumors from mice dosed with ^99m^Tc-225.28 (20.0 ± 6.7% ID/g) was higher than in any other organ and was significantly higher than that in tumors from mice dosed with ^99m^Tc-F3-C25 (8.5 ± 2.6% ID/g) (*p* < 0.05) (Figure 3). The ^99m^Tc activity in these tumors (20.0 ± 6.7% ID/g) was similar to the ^212^Pb activity (19.0 ± 9.2% ID/g) observed in the SUM159 xenografts in biodistribution study 2 with ^212^Pb-225.28 (Figure 2A). Most normal organs from both ^99m^Tc-225.28 and ^99m^Tc-F3-C25 dose groups showed a comparable uptake of ^99m^Tc activity (Figure 3). The livers, spleens, kidneys, and femurs from mice dosed with the ^99m^Tc-RICs showed lower uptake of the injected activity compared to the mice dosed with the ^212^Pb-RICs in the other biodistribution studies. The collective biodistribution results indicated (a) different radionuclides influenced the normal tissue distribution and RES retention of the RICs tested in these studies, (b) tumor size had a greater influence than the choice of radionuclide on the specific uptake of radiolabeled mAb 225.28 in established SUM159 xenografts, and (c) the uptake of activity from ^212^Pb-225.28 in TNBC xenografts correlates with relative in vitro epitope expression on the tumor cells.

### 2.4. ^212^Pb-225.28 Inhibits the Growth of Human TNBC Xenografts

**Figure 4:**
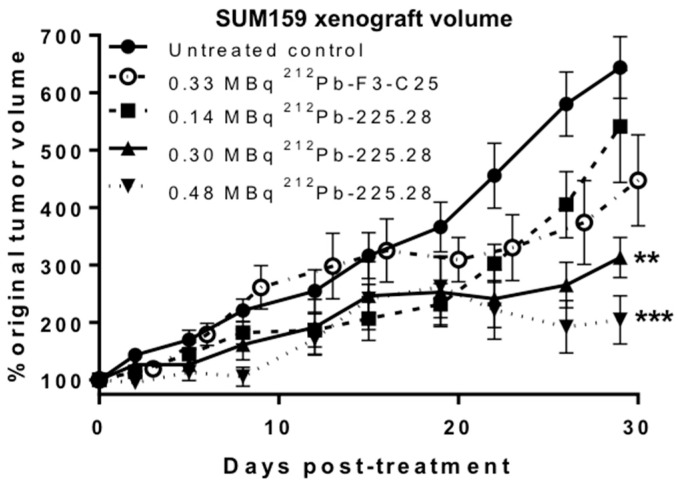
In vivo tumor growth curves showing the effects of ^212^Pb-225.28 or ^212^Pb-F3-C25 on human TNBC xenograft growth inhibition. Groups of mice (*n* = 10/group) bearing small SUM159 tumors either were left untreated (filled circles) or injected *i.v.* with 0.33 MBq ^212^Pb-F3-C25 (open circles) or with 0.14 MBq (filled squares), 0.30 MBq (upward triangles), or 0.48 MBq (downward triangles) ^212^Pb-225.28. Data were plotted as mean percent change (±standard error of the mean) in normalized tumor volume over time relative to the initial volume at the time of injection with the RIC, and analyzed by one-way ANOVA with Bonferroni’s comparison. ** *p* < 0.01 or *** *p* < 0.001 vs. ^212^Pb-F3-C25 at day 29 after dosing.

An in vivo tumor growth inhibition study was initiated in groups of mice bearing small (mean 73 mm^3^) SUM159 xenografts in the mammary fat pad when tumors had not yet reached the logarithmic stage of growth. An early time point of xenograft development was chosen for this RIT study due to the known challenge of eradicating larger, solid tumors with α-particle RICs [31,32,33]. Groups of mice were given a single *i.v.* dose at different radionuclide levels of ^212^Pb-F3-C25 or ^212^Pb-225.28; one group of mice received no treatment. As shown in Figure 4, all doses of ^212^Pb-225.28 inhibited SUM159 xenograft growth compared to non-treated controls within the first 7–12 days of dosing. A dose-dependent effect on tumor growth inhibition became apparent by three weeks after dosing in mice treated with 0.14, 0.30, or 0.48 MBq ^212^Pb-225.28; this effect was significant at day 29 after dosing (*p* < 0.05). Tumor growth was inhibited to a significantly greater extent (*p* < 0.01) in the groups of mice given 0.30 or 0.48 MBq ^212^Pb-225.28 compared to the group given 0.33 MBq ^212^Pb-F3-C25 at day 29 after dosing. No complete tumor regressions were observed in these studies. All groups of mice given the ^212^Pb-RICs showed a transient dose-related drop in body weight ranging from 3–10% within the first week after dosing (Supplementary Figure S3). All groups recovered to their initial body weight within 3–4 weeks of dosing, although mice in the groups given 0.30 or 0.48 MBq ^212^Pb-225.28 had lower weights than control mice throughout the study. Two mice and four mice in the groups that received 0.30 or 0.48 MBq ^212^Pb-225.28, respectively, died at 11–12 days after dosing. No other adverse effects on mouse health were noted in these RIT efficacy proof-of-concept studies.

## 3. Discussion

Patients with TNBC have higher rates of metastatic and chemotherapy-resistant recurrent disease, resulting in lower survival rates compared to other subtypes of breast cancer [1,2]. Developing novel therapies that specifically target and kill both differentiated TNBC cells and CICs without causing unacceptable systemic toxicity would help to improve the prognosis for patients with TNBC. Previous studies have shown that CSPG4 is overexpressed on the majority of TNBC differentiated cells and CICs relative to most normal tissues [28,29,34]. The studies presented here aimed to determine if CSPG4 represents a useful marker and target to monitor and destroy TNBC tumors. To this end, CSPG4-specific mAb 225.28, radiolabeled with diagnostic and therapeutic radionuclides, was tested for its ability to bind to and halt the proliferation of preclinical models of human TNBC. Performing these studies was an essential first step toward demonstrating the concept that RICs of mAb 225.28 could successfully be used for CSPG4-targeted α-particle RIT of TNBC. The daughter α-particle emitters generated during the decay of ^212^Pb emit short-range, high LET radiation that can effectively kill tumor cells. Conjugating ^212^Pb to mAb 225.28 was intended to mediate the specific uptake and retention of the radionuclides in CSPG4-expressing TNBC tumors while sparing the majority of normal tissues which do not express CSPG4 from the harmful effects of α-particle radiation.

The in vitro binding and clonogenic survival studies were used to characterize the binding affinity of the CSPG4-targeted ^212^Pb-RIC, to quantify the expression of the CSPG4 epitope on human TNBC cells and to demonstrate the efficacy of CSPG4-targeted ^212^Pb RIT at inhibiting TNBC cell proliferation. The results from these studies showed that mAb 225.28 labeled with ^212^Pb maintained its affinity and specificity for the target CSPG4 epitope and mediated retention of the radionuclides in human TNBC differentiated cells and CICs. Relative to adherent SUM159 cells, the SUM159 CICs showed higher numbers of binding sites per cell and a higher sensitivity to ^212^Pb-225.28. This result is consistent with modeling and experimental studies, showing that cells with higher antigen expression and occupancy with α-particle RICs are subjected to higher dose rates, resulting in lower cell proliferative potential [12,15,35]. The lower clonogenic survival of the CICs compared to adherent SUM159 cells is also likely due to the penetration of α-particles into the center of the mammospheres following decay of ^212^Pb-RICs bound to the outer layer of cells on the mammospheres. The relative sensitivity of the SUM159 cells to the CSPG4-targeted ^212^Pb-RIC compared to the control ^212^Pb-RIC in these studies is consistent with previous results using other breast cancer cell lines and α-particle RICs [15,21], thus indicating mAb 225.28 is a suitable carrier for targeted RIT of this TNBC tumor model.

The biodistribution studies were used to measure the retention of the CSPG4-targeted RICs in animal models of human TNBC. The results from the three sets of biodistribution studies confirmed that the CSPG4-specific mAb 225.28 RICs specifically bound to the target antigen in established TNBC xenografts in vivo. Comparing biodistribution study 1 to study 2 shows that ^212^Pb-225.28 accumulated differently in small vs. larger SUM159 solid tumor xenografts. This result suggests there may be a threshold of TNBC tumor development for optimal targeting with mAb 225.28. Biodistribution study 2 showed that higher tumor uptake of ^212^Pb-225.28 occurred in SUM159 tumors relative to 2LMP tumors, suggesting that the relative number of binding sites on the two TNBC cell lines, as determined in the in vitro studies (Table 1), influenced the specific retention of the RIC in TNBC tumors in vivo. This result is consistent with previous receptor-targeted studies that have used radiolabeled molecules that bind to alternative targets [36,37,38,39]. Detailed investigations to identify further causes for the observed differences in RIC accumulation in the various TNBC xenografts, such as histological analyses of CSPG4 epitope expression or vasculature formation at different time points of tumor development, were beyond the scope of the studies presented here and are worthy of future examination.

Comparing biodistribution studies 1 and 2 to study 3 shows that the different radiolabeling conditions used with mAb 225.28 affected the uptake of the RIC in SUM159 tumors and in normal organs. Variability in tissue distribution between RICs with different radionuclides was anticipated based on the labeling conditions, chelating agents, and known tissue retention for the different radionuclides used [40,41]. The number of TCMC chelates per mAb and potential radiolysis from the α-particles likely caused recognition of the ^212^Pb-RICs by the RES and contributed to their removal from circulation [42,43]. The specific mice used in the present studies showed particularly avid RES uptake of the ^212^Pb-RICs, compared to parallel biodistribution studies in nude mice from an alternative supplier that showed less RES uptake of the same batch of ^212^Pb-F3-C25 that was used in the studies above [33]. The collective biodistribution results are consistent with previous studies that used an alternative IgG_2a_ mAb (376.96) labeled with ^212^Pb in mice, as well as studies in rodents and humans that showed accumulation of radioactivity in the kidneys, spleens, livers, and bones after injection of CSPG4-targeted RICs [44,45,46,47]. The diagnostic radionuclide ^99m^Tc (*t*_1/2_ = 6.02 h; 140 keV, 89%) was used in biodistribution study 3 due to its availability within the intended timeframe of tumor development and to its defined radiochemistry and favorable gamma energy for imaging. CSPG4 antibodies could be labeled with ^203^Pb (*t*_1/2_ = 51.9 h; 279.2 keV, 80.9%) and ^212^Pb as an alternative matched-pair for diagnostic imaging and RIT.

The purpose of the initial therapy study was to test if a CSPG4-targeted ^212^Pb-RIC could halt the proliferation of established xenografts of human TNBC in vivo. The results from this study showed that ^212^Pb-225.28 mediated significant, specific, dose-dependent inhibition of SUM159 xenograft growth in mice. Importantly, significantly greater tumor growth inhibition from ^212^Pb-225.28 (0.30 MBq) was observed relative to a slightly higher dose of ^212^Pb-F3-C25 (0.33 MBq), demonstrating the efficacy and specificity of the CSPG4-targeted RIC against an in vivo model of human TNBC. The lack of complete tumor regression indicated that the dose levels and timing of administration of ^212^Pb-225.28 explored in these studies did not eliminate all proliferating cells in the TNBC xenografts. The moderate therapeutic efficacy is consistent with the results from biodistribution study 1 showing relatively low uptake of the injected ^212^Pb activity in the small SUM159 xenografts. While dosimetry studies were beyond the scope of these proof-of-concept studies, dosimetry analyses at multiple time points after injection of ^212^Pb-225.28 would contribute to predicting the likelihood of tumor control when using this RIC. Dose escalation would likely not expand the therapeutic window, as acute toxicity (transient drops in body weights) and deaths of mice within 12 days of dosing with 0.30 MBq or more of the ^212^Pb-RICs indicated the maximum tolerated dose was reached in these studies. The radiochemical purity (<80%) of ^212^Pb-225.28 used in this particular therapy study likely influenced the accumulation of the radionuclides in normal tissues and contributed to systemic toxicity, since mice dosed with 0.33 MBq ^212^Pb-F3-C25, which had a higher radiochemical purity (~95%), recovered weights faster than mice given a slightly lower dose of ^212^Pb-225.28 (0.30 MBq) and had comparable weights to the non-treated control group within two weeks of dosing. Tissue histology and toxicology analyses to identify affected organs were beyond the scope of these initial RIT efficacy studies, although the biodistribution results suggest RES tissues (e.g., spleen, liver) and blood cell counts would be worthy of examination for toxicology in future studies. Previous studies with alternative α-particle RICs have shown that higher uptake of the RIC in the tumor lowers the dosimetry to normal tissues and corresponds to greater tolerability [48]. Future studies that include multiple time points for biodistribution, dosimetry, and toxicology analyses with ^212^Pb-225.28 would aid the interpretation of therapeutic efficacy, tolerability, and optimization of the therapeutic window with CSPG4-targeted RICs.

The results from the above in vivo studies are consistent with prior experiments utilizing other α-particle RICs against established breast tumors in mice. A ^213^Bi-labeled RIC targeting HER-2/*neu* receptors did not eradicate tumors but was more effective than ^213^Bi-labeled control RICs at prolonging survival and inhibiting tumor growth in subcutaneous and metastatic breast cancer models [19]. Utilizing a longer-lived radionuclide, ^225^Ac (*t*_1/2_ = 10.0 d), in the HER-2/*neu*-targeted RIC at its maximum tolerated dose prevented metastatic breast tumor formation in over half of the treated mice, while the irrelevant control, ^225^Ac-RIC, had no effect against metastatic tumor development [21]. Biodistribution and dosimetry analyses using an anti-PD-L1 ^225^Ac-RIC in immune-competent mice with syngeneic, solid mammary tumors showed heterogeneous distribution of intra-tumoral activity [26]. While therapeutic efficacy was not determined for the ^225^Ac-RIC, calculations indicated that changing the radionuclide to ^212^Pb would decrease the absorbed dose in the tumor due to the shorter half-life and lower cumulative energy emitted in the decay chain of ^212^Pb compared to ^225^Ac [26]. Studies with ^227^Th-trastuzumab showed reduced HER-2^+^ breast xenograft growth relative to non-treated controls; dose fractionation failed to improve therapeutic outcome compared to a single administration of the RIC [48]. As observed in the present study, these prior studies showed that the time interval between tumor cell inoculation and administering the RICs influenced biodistribution and therapeutic outcomes, where earlier initiation of therapy after tumor cell implantation improved therapeutic outcomes [19,21,48].

Limitations in tissue extravasation, radionuclide half-life, and specific activity are barriers to eliminating solid tumors with intact mAbs as carrier molecules for targeted α-particle RIT. Utilizing smaller mAb fragments (e.g., scFv, F(ab′)_2_, scFv-Fc) that target CSPG4 [46,49] could be employed to improve tumor penetration and normal tissue clearance kinetics, and thus enhance therapeutic efficacy and tolerability. CSPG4-targeted RIT in combination with approved chemotherapy or novel molecular inhibitors of CIC proliferation are also worthy of future investigation. While primary TNBC tumors can be resected, the elimination of metastases or recurrent tumors from refractory differentiated cells and CICs that survive current therapies remains a significant clinical challenge in TNBC. Further studies using animal models with pre-vascular malignant lesions or metastases are anticipated to be more relevant than solid tumor xenografts in assessing the therapeutic potential of α-particle RICs against TNBC. The expression of the CSPG4 antigen in other malignancies [4,5,28,29] suggests ^212^Pb-225.28 would also be worthy of evaluation for targeted α-particle RIT of additional types of cancer in future studies.

Additional dosimetry and toxicology studies in animal models would be required prior to clinical assessment of CSPG4-targeted α-particle RIT. Humanization of mAb 225.28 is necessary prior to multi-fraction treatments with ^212^Pb-225.28 in human studies to avoid human anti-mouse antibody clearance of the RIC from the circulation. A CSPG4-specific fully human single-chain antibody is available [49] that could be labeled with ^212^Pb for future clinical RIT studies. Clinical treatment with CSPG4-targeted ^212^Pb-RICs would be relevant for adjuvant settings after resection of the primary macroscopic tumor, or in treating early stages of recurrent or metastatic disease, when microscopic populations of malignant cells are present. In these situations, the α-particle emitting RICs bound to the cells could halt further growth of the disease. The ability of CSPG4-specific ^212^Pb-225.28 to target and inhibit the growth of subcutaneous TNBC xenografts in mouse models supports the concept of assessing its efficacy against metastatic TNBC models prior to clinical testing [28].

## 4. Materials and Methods

### 4.1. Reagents and Instrumentation

All reagents were prepared from commercially available materials (Thermo Fisher, Waltham, MA, USA; Sigma, St. Louis, MO, USA) unless specified otherwise. ^212^Pb in transient equilibrium with its daughter radionuclides was eluted as previously described [50] from a ^224^Ra/^212^Pb generator (Oak Ridge National Laboratory, Oak Ridge, TN, USA). Murine mAb 225.28 [28,30] and isotype-matched control murine mAb F3-C25 that is anti-idiotypic to a murine anti-HLA Class II mAb [51] were produced and characterized as previously described. A calibrated high-purity germanium (HPGe) detector (model GMX10P4-70; Ortec, Oak Ridge, TN, USA), operated at −4000 V, housed in a lead shield (Ortec, Oak Ridge, TN, USA), was used to determine the radionuclidic purity and radioactivity of ^212^Pb for all radiolabeling procedures. Spectra were processed using Gamma Vision-32 software (version 6.09; Ortec). Radioactivity measurements of samples from the in vitro and in vivo experiments were performed on calibrated Cobra II (Packard, Meriden, CT, USA) or Wizard^2^ (Perkin Elmer, Shelton, CT, USA) gamma counters using an energy window centered on the main gamma peak from ^212^Pb (238.6 keV, 43.6%) after cross-calibrating the instruments with the HPGe detector. Radioactivity analyses were corrected for radioactive decay.

### 4.2. Radiolabeling

mAbs 225.28 and F3-C25 were separately conjugated with the bifunctional chelator, 2-(4-isothiocyanotobenzyl)-1,4,7,10-tetraaza-1,4,7,10-tetra-(2-carbamoylmethyl)-cyclododecane (TCMC; Macrocyclics, Plano, TX, USA), following previously described procedures [52] to generate TCMC-mAb 225.28 and TCMC-mAb F3-C25. The average chelate/mAb ratios were determined by a spectrophotometric assay [53]. Radiolabeling and purification of the ^212^Pb-TCMC-mAb radioimmunoconjugates (RICs), ^212^Pb-225.28 or ^212^Pb-F3-C25, were performed as previously described [50] using 0.11–0.86 MBq of ^212^Pb per 1 μg of TCMC-mAb conjugate (17–130 GBq/μmol). Immediately after collecting the purified RICs, an aliquot was removed for additional characterization and 5 μL of 0.1 mol/L EDTA and 50 μL 30% human serum albumin (Sigma-Aldrich, St. Louis, MO, USA) were added to the RICs. The protein content of the removed aliquot was determined by Lowry analysis [54]. The radiochemical conversions and purities of the crude preparations and isolated RICs were determined as previously described [50]. mAb 225.28 containing 6.1 TCMC chelates per mAb was radiolabeled with ^212^Pb to produce ^212^Pb-225.28 in an average yield of 61% and a radiochemical purity of 85% (*n* = 6), as indicated by ITLC analysis. The control mAb F3-C25 containing 5.6 TCMC chelates per mAb molecule was radiolabeled to give ^212^Pb-F3-C25 with an average radiochemical purity of 95% (*n* = 5). Specific activities of 46–260 kBq/μg (6.8–40 GBq/μmol) for the ^212^Pb-RICs were used in the in vitro and in vivo studies described below. TCMC-mAb 225.28 and TCMC-mAb F3-C25 were separately conjugated with HYNIC-NHS and radiolabeled with ^99m^Tc-tricine, as previously described [55]; the isolated specific activity was 0.98 MBq/μg (91% radiochemical purity) for ^99m^Tc-225.28 and 1.03 MBq/μg (90% radiochemical purity) for ^99m^Tc-F3-C25. mAb F3-C25 was radiolabeled with carrier-free Na^125^I (MP Biomedicals, Solon, OH, USA) in Pierce Pre-Coated Iodination Tubes (Thermo Scientific, Rockford, IL, USA) according to the manufacturer’s specifications; the isolated specific activity was 24.2 kBq/μg (91% radiochemical purity). The resulting ^125^I-F3-C25 solution was stored in 5 mg/mL bovine serum albumin in PBS at 4 °C.

### 4.3. Human TNBC Cell Lines and CICs

The human TNBC cell lines, SUM159 and 2LMP, were cultured under adherent conditions, as previously described [56]. Nonadherent mammospheres (“CIC” conditions) were cultured with MEGM medium (Lonza, Walkersville, MD, USA) in ultra-low attachment plates (Corning Costar, Corning, NY, USA). After 3 days of culture in CIC conditions, SUM159 mammospheres grew as irregular clusters (60–120 μm) of tightly joined cells; 2LMP mammospheres grew as non-uniform aggregates (90–230 μm) of cells. Mammospheres from both cell lines maintained high viability (>95%) under these conditions. Cell culture and in vitro experiments were performed at 37 °C in a 5% CO_2_ humidified atmosphere unless specified otherwise.

### 4.4. In Vitro Binding Assays

Binding assays with serial dilutions (5 to 0.03 nM mAb concentration) of ^212^Pb-225.28 to SUM159 or 2LMP adherent cells and CICs (dissociated from mammospheres with Cell Stripper [Corning]) were performed as previously described with an alternative ^212^Pb-RIC and ovarian cancer cell lines, to calculate the binding affinity (*K*_d_), binding sites per cell, and internalized fraction [47,55].

### 4.5. In Vitro Clonogenic Survival Assays

Adherent clonogenic assays: SUM159 cells were seeded at 10,000 cells/well in 24-well plates (Corning Costar) two days before treatment with the RICs, defined as day 0 of the study. On day 0, cells were rinsed with PBS and incubated with serial dilutions (1095 to 0.8 kBq/mL ^212^Pb concentration) of ^212^Pb-225.28 or ^212^Pb-F3-C25 in 0.4 mL assay medium (DMEM pH 7.4 with 30 mmol/L HEPES, 2 mmol/L l-glutamine, 1 mmol sodium pyruvate, 1% bovine serum albumin) for 2 h at 37 °C with gentle swirling. Cells were rinsed twice with PBS and incubated in fresh Adherent medium. Two days later (day 2), cells were trypsinized, counted, serially diluted in normal (Adherent) medium, and plated at 100 live cells/well in six replicate wells of a tissue culture treated 6-well plate (Corning Costar). The percent clonogenic survival relative to vehicle-treated controls (set to 100% survival) was determined 8 days after plating (day 10), as previously described [47,57]. The maximal response (no survival) at the highest concentration of ^212^Pb tested (1095 kBq/mL) was set to 0% survival.

CIC clonogenic assays: SUM159 cells were seeded at 100,000 cells/well in 24-well ultra-low attachment plates in CIC conditions to form mammospheres three days before treatment with RICs, defined as day 0 of the study. On day 0, intact mammospheres were collected, rinsed with PBS, and incubated with serial dilutions (1095 to 0.8 kBq/mL ^212^Pb concentration) of ^212^Pb-225.28 or ^212^Pb-F3-C25 in 0.4 mL assay medium for 2 h at 37 °C with gentle swirling to keep mammospheres in suspension. Mammospheres were rinsed twice with PBS and incubated in new ultra-low attachment 24-well plates with fresh CIC medium. Two days later (day 2), mammospheres were collected and dissociated into single cells with Accutase or trypsin, counted, and serially diluted in normal (Adherent) medium. Cells were plated and analyzed for clonogenic survival as in the Adherent clonogenic assays.

### 4.6. Animal Subjects and Husbandry

All in vivo studies were performed in 5–10 week old female athymic nude mice (Envigo, Indianapolis, IN, USA). The University of Alabama at Birmingham Institutional Animal Care and Use Committee reviewed and approved animal studies before the studies were initiated (09722; updated approval 25 October 2016). Studies were performed in compliance with guidelines from the Public Health Service Policy and Animal Welfare Act of the United States. Single-cell suspensions of SUM159 or 2LMP cells (4 × 10^6^), mixed 1:1 with Matrigel, were implanted in the mammary fat pads of mice. Radioactive biodistribution studies were initiated at 7, 11, or 14 days after implantation; therapy studies were initiated at 6 or 7 days after implantation. Mice in therapy studies were given a Nutra-Gel Diet (Bio-Serv, Flemington, NJ, USA) in addition to standard chow for two weeks after administering the RICs. Mice were weighed twice per week and euthanized if >10% weight loss occurred or when tumors grew to 1.5 cm in diameter. Tumor volume was calculated by the formula (L × W^2^)/2 [31].

### 4.7. In Vivo Biodistribution and Imaging Studies

Biodistribution study 1: At day 7 after tumor cell implantation, mice bearing small (67 ± 6 mm^3^) SUM159 xenografts (*n* = 4 mice/group) were injected *i.v*. with ~0.78 MBq ^212^Pb-225.28 (6.2 μg) or ^212^Pb-F3-C25 (4.5 μg) in 0.2 mL PBS. Mice were euthanized at 24 h post injection, when selected tissues were collected, weighed, and counted with a gamma counter. Percent uptake of the injected dose per gram (% ID/g) was calculated by comparing the tissue activity to solutions with known activity of the radionuclide of interest.

Biodistribution study 2: At day 11 after tumor cell implantation, mice bearing large (~120 mm^3^) SUM159 or 2LMP xenografts (*n* = 5 mice/group) were injected *i.v.* with a solution containing ^212^Pb-225.28 (0.68 MBq, 4.1 μg) and ^125^I-F3-C25 (16.3 kBq, 4.2 μg) in 0.2 mL PBS. Mice were euthanized at 24 h post injection, and selected tissues were removed, weighed, and counted to determine the % ID/g as above. ^125^I was counted after ^212^Pb had fully decayed.

Imaging and biodistribution study 3: At day 14 after tumor cell implantation, mice bearing medium–large (94 ± 17 mm^3^) SUM159 xenografts (*n* = 3–4 mice/group) were injected *i.v*. with 6.1 MBq (~6 μg) ^99m^Tc-225.28 or ^99m^Tc-F3-C25 in 0.2 mL PBS. At 23 h after injection, mice were anesthetized with isoflurane and imaged for 15 min on a dual-head planar gamma camera (X-SPECT, Gamma Medica-Ideas, Northridge, CA, USA) with a 1 mm low-energy pinhole collimator. Mice were euthanized at 24 h post injection, when tissues were collected, weighed, and counted to determine the % ID/g as above.

### 4.8. In Vivo Therapy Studies

Groups of mice (*n* = 10/group) bearing small (73 ± 4 mm^3^) SUM159 xenografts were given a single *i.v*. dose (0.14–0.48 MBq) of ^212^Pb-225.28 or of ^212^Pb-F3-C25 or received no treatment (control). Mice in the ^212^Pb-225.28 groups were dosed at day 7 after tumor cell implantation; mice in the ^212^Pb-F3-C25 group were dosed at day 6 after tumor cell implantation. Tumor growth was calculated as the mean percent change in volume with standard error, relative to the initial volume at the time of dosing.

### 4.9. Statistical Analyses

Data were analyzed using Microsoft Excel or GraphPad Prism (Version 5.02, GraphPad Software, La Jolla, CA, USA). *K*_d_ values were calculated from the total and non-specific Scatchard binding data by fitting to a one-site binding curve using non-linear regression. IC_50_ values were calculated from the clonogenic survival data by fitting with non-linear regression to a variable slope dose-response inhibition curve. Student’s *t*-test was used when comparing two groups. When comparing multiple groups, one-way ANOVA tests, followed by Bonferroni corrections for multiple comparisons, were performed. All *p*-values correspond to two-tailed tests; significance was considered to be at *p* < 0.05, unless required Bonferroni corrections were applied for multiple comparisons.

## 5. Conclusions

^212^Pb-225.28 bound with high affinity to CSPG4 expressed on TNBC differentiated cells, CICs, and xenograft tumors in mice. Different cell lines of TNBC displayed varying levels of the target epitope in vitro, which correlated with the in vivo uptake of ^212^Pb-225.28 in the respective TNBC xenografts. RICs of mAb 225.28 showed a favorable, specific uptake in actively growing TNBC xenografts; non-targeted RES uptake was higher for the ^212^Pb-labeled RICs compared to RICs labeled under less radiolytic conditions. ^212^Pb-225.28 specifically inhibited the growth of TNBC xenografts with moderate toxicity at therapeutically effective doses. These results support future dosimetry, toxicology, and therapy studies using ^212^Pb-225.28 or other CSPG4-targeted RICs, alone or in combinatorial therapy approaches against models of TNBC.

## Figures and Tables

**Table 1:** In vitro binding analysis of ^212^Pb-225.28 to human triple-negative breast cancer (TNBC) cells and cancer-initiating cells (CICs).

	^a^*K*_d_ ± S.E.M. (nmol/L)	^a^ Binding Sites/Cell ± SEM (×10^3^)	Internalization (%)
Adherent SUM159	0.6 ± 0.1	29 ± 4.2	56
CIC SUM159	0.3 ± 0.1	49 ± 12	57
Adherent 2LMP	0.5 ± 0.1	^b^ 3.4	49
CIC 2LMP	0.5 ± 0.2	^b^ 7.7	47

^a^ Data are presented as the mean ± SEM of 1–3 individual experiments, with each experiment performed with duplicate wells. ^b^ Mean of a single experiment performed with duplicate wells.

**Table 2:** In vitro inhibition of SUM159 cells and CIC clonogenic survival by ^212^Pb-225.28 or ^212^Pb-F3-C25.

Cells	^a^ IC_50_ ± SEM (kBq/mL)	
	^212^Pb-225.28	^212^Pb-F3-C25
Adherent SUM159	22 ± 10	144 ± 19
CIC SUM159	12 ± 3	86 ± 10

^a^ Data are presented as the mean ± SEM of two individual experiments, with each experiment performed with 4–6 replicate wells.

## Supplementary Materials

Supplementary materials can be found at http://www.mdpi.com/1422-0067/19/4/925/s1.

## Abbreviations

TNBC	Triple negative breast cancer
CIC	Cancer initiating cell
RIT	Radioimmunotherapy
CSPG4	Chondroitin sulfate proteoglycan 4
mAb	Monoclonal antibody
RIC	Radioimmunoconjugate
HER-2	Human epidermal growth factor receptor 2
LET	Linear energy transfer
keV	Kiloelectron volt
*i.v.*	Intravenous
HPGe	High purity germanium
TCMC	2-(4-isothiocyanotobenzyl)-1,4,7,10-tetraaza-1,4,7,10-tetra-(2-carbamoylmethyl)-cyclododecane
EDTA	Ethylene diaminetetraacetic acid
ITLC	Instant thin layer chromatography
ANOVA	Analysis of variance
RES	Reticuloendothelial system
