# Supplementary material for: 212Pb-Labeled Antibody 225.28 Targeted to Chondroitin Sulfate Proteoglycan 4 for Triple-Negative Breast Cancer Therapy in Mouse Models
Source: Int J Mol Sci. 2018 Mar 21;19(4):925. doi: 10.3390/ijms19040925 (PMC5979285; doi:10.3390/ijms19040925)
Supplement: Supplementary file 1 [file ijms-19-00925-s001.pdf]

International Journal of
Molecular Sciences

MDPI

Supplementary

^{212}Pb-Labeled Antibody 225.28 Targeted to Chondroitin Sulfate Proteoglycan 4 for Triple-Negative Breast Cancer Therapy in Mouse Models

Benjamin B. Kasten, Patsy G. Oliver, Harrison Kim, Jinda Fan, Soldano Ferrone, Kurt R. Zinn and Donald J. Buchsbaum

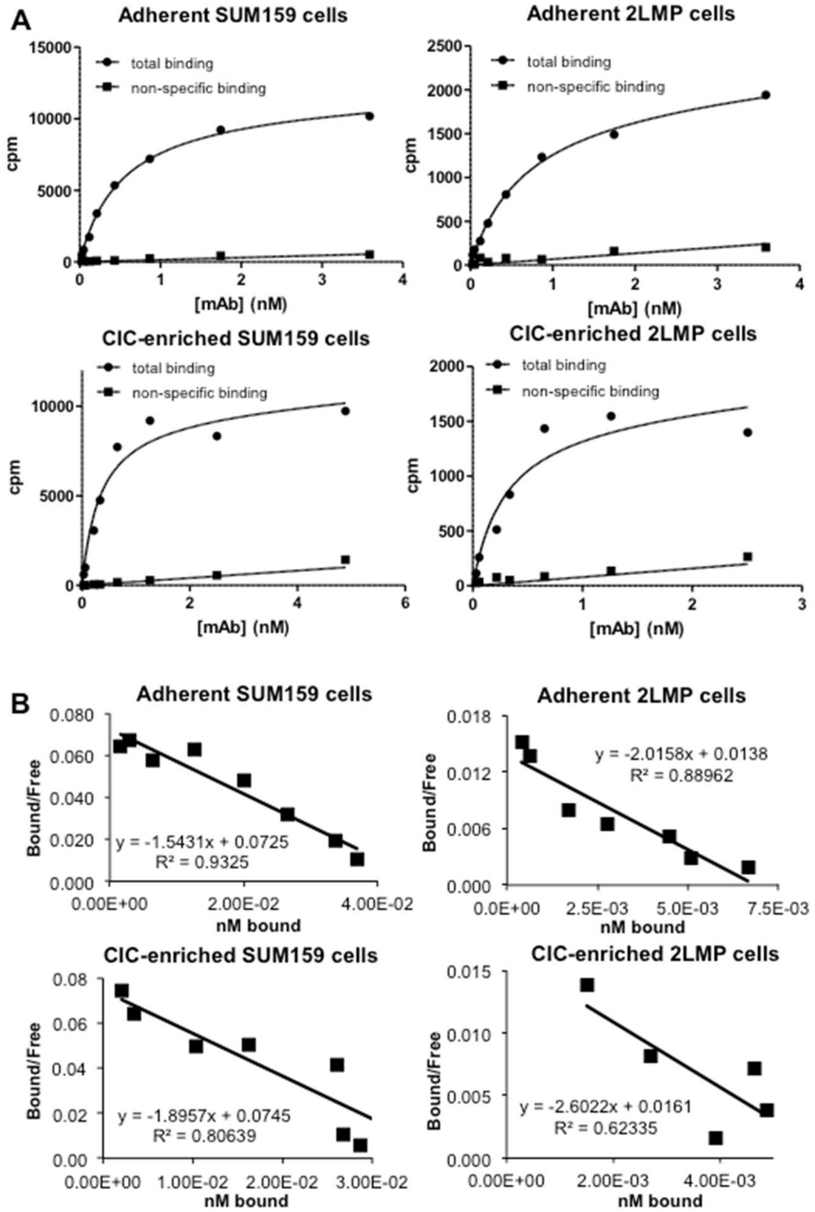

Figure S1. Representative graphs showing the in vitro binding results of ^{212}Pb-225.28 to the surface of human TNBC cells and CICs (**A**) and Scatchard plots of the data (**B**).

Table S1. Results from biodistribution study 1, showing the %ID/g of ^{212}Pb at 24 h after *i.v.* injection of ^{212}Pb-225.28 or ^{212}Pb-F3-C25 in athymic nude mice bearing small (mean 67 mm^3) SUM159 xenografts.

	**^{212}Pb-225.28**		**^{212}Pb-F3-C25**	
**Tissue**	**Mean % ID/g**	**Standard deviation % ID/g**	**Mean % ID/g**	**Standard deviation % ID/g**
Heart	2.7	0.8	2.4	0.2
Liver	17.6	6.4	30.0	1.8
Stomach	0.5	0.2	0.5	0.5
Large Intestine	1.8	0.6	1.9	1.7
Small Intestine	4.5	1.8	2.9	0.9
Cecum	1.4	0.7	1.2	0.7
Spleen	29.2	17.7	43.5	15.0
Lungs	3.6	2.1	1.8	0.1
L Kidney	10.2	2.1	8.9	0.3
R Kidney	10.8	1.8	10.3	1.8
Leg Muscle	0.8	0.6	0.4	0.1
Blood	4.9	5.0	1.7	0.2
Rep Organs	3.8	1.4	2.8	0.8
Brain	0.2	0.2	0.1	0.0
Femur	4.3	0.6	10.3	4.3
Tumor	8.3	5.7	3.0	0.7

Table S2. Results from biodistribution study 2 showing the %ID/g of the respective radionuclide (^{212}Pb or ^{125}I) at 24 h after *i.v.* co-injection of ^{212}Pb-225.28 and ^{125}I-F3-C25 in athymic nude mice bearing large (mean ~120 mm^3) SUM159 xenografts.

	**^{212}Pb-225.28**		**^{125}I-F3-C25**	
**Tissue**	**Mean % ID/g**	**Standard deviation % ID/g**	**Mean % ID/g**	**Standard deviation % ID/g**
Heart	3.7	0.5	2.7	0.6
Liver	13.5	4.9	3.4	0.5
Stomach	0.9	0.4	3.0	1.2
Large Intestine	2.2	0.7	1.4	0.4
Small Intestine	3.6	2.0	1.8	0.7
Cecum	1.8	0.5	1.1	0.2
Spleen	22.3	17.5	5.7	4.3
Lungs	6.2	1.8	4.9	1.3
L Kidney	9.9	1.1	3.1	0.2
R Kidney	9.8	0.4	3.0	0.3
Leg Muscle	1.4	0.6	1.2	0.4
Blood	11.2	5.5	10.4	3.7
Rep Organs	6.8	3.5	4. 1	1.7
Brain	0.4	0.2	0.3	0.1
Femur	4.6	3.3	2.1	0.4
Tumor	19.0	9.2	4.7	1.7

Table S3. Results from biodistribution study 2 showing the %ID/g of the respective radionuclide (^{212}Pb or ^{125}I) at 24 h after *i.v.* co-injection of ^{212}Pb-225.28 and ^{125}I-F3-C25 in athymic nude mice bearing large (mean ~120 mm^3) 2LMP xenografts.

	**^{212}Pb-225.28**		**^{125}I-F3-C25**	
**Tissue**	**Mean % ID/g**	**Standard deviation % ID/g**	**Mean % ID/g**	**Standard deviation % ID/g**
Heart	2.9	0.9	2.1	0.6
Liver	15.9	6.3	3.9	1.0
Stomach	0.9	0.5	2.6	1.2
Large Intestine	2.0	1.0	1.2	0.5
Small Intestine	5.8	4.4	2.5	1.7
Cecum	1.7	0.7	1.0	0.4
Spleen	26.1	17.1	7.3	5.0
Lungs	4.2	2.3	3.5	1.3
L Kidney	8.5	1.6	2.2	0.4
R Kidney	9.0	2.0	2.4	0.6
Leg Muscle	1.0	0.6	0.8	0.3
Blood	7.7	7.6	8.0	5.0
Rep Organs	4.0	2.4	2.5	1.3
Brain	0.2	0.2	0.2	0.2
Femur	6.4	3.1	2.7	0.8
Tumor	9.7	4.9	4.0	1.1

Table S4. Results from biodistribution study 3 showing the %ID/g of ^{99m}Tc at 24 h after *i.v.* injection of ^{99m}Tc-225.28 or ^{99m}Tc-F3-C25 in athymic nude mice bearing medium–large (mean 94 mm^3) SUM159 xenografts.

	**^{99m}Tc-225.28**		**^{99m}Tc-F3-C25**	
**Tissue**	**Mean % ID/g**	**Standard deviation % ID/g**	**Mean % ID/g**	**Standard deviation % ID/g**
Heart	2.7	0.7	3.2	1.0
Liver	10.1	4.2	10.6	2.3
Stomach	1.0	0.3	1.2	0.5
Large Intestine	2.0	0.2	2.3	0.7
Small Intestine	4.7	3.5	4.8	3.3
Cecum	1.9	0.6	2.6	0.5
Spleen	11.2	4.1	9.9	3.7
Lungs	4.0	1.6	5.5	1.5
L Kidney	5.4	1.0	5.3	1.1
R Kidney	5.4	1.1	5.6	0.8
Leg Muscle	0.6	0.2	1.2	0.5
Blood	5.9	4.0	8.9	5.6
Rep Organs	3.4	1.3	5.0	1.2
Brain	0.2	0.2	0.3	0.2
Femur	2.7	1.2	2.3	0.7
Tumor	20.0	6.7	8.5	2.6

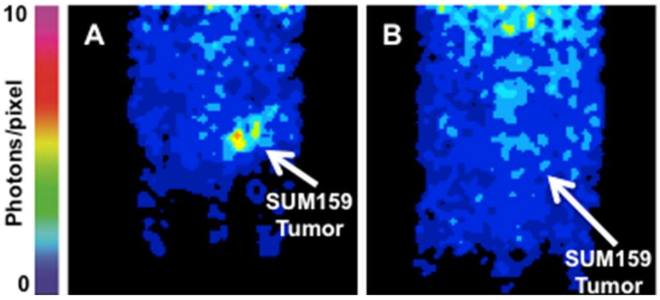

Figure S2. In vivo imaging of ^{99m}Tc-RICs in mice bearing medium–large (mean 94 mm^3) orthotopic SUM159 xenografts. Representative in vivo planar gamma camera images at 23 h after *i.v.* injection of 6.1 MBq ^{99m}Tc-225.28 (**A**) or ^{99m}Tc-F3-C25 (**B**) show ^{99m}Tc activity in the region of the tumor. Mice were anesthetized by isoflurane during imaging. The images correspond to a 4.11 cm field of view and are scaled to the same intensity.

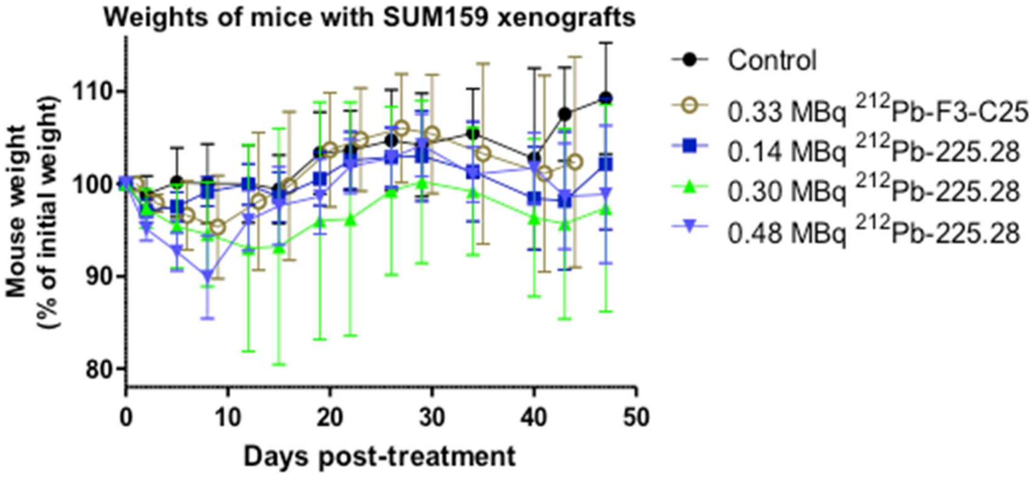

Figure S3. Effects of treatment on weights of athymic nude mice bearing small (mean 73 mm^3) SUM159 xenografts during in vivo therapy studies with ^{212}Pb-225.28 or ^{212}Pb-F3-C25. Data were plotted as the mean percent change in weight (± standard deviation) over time relative to the normalized weight at the time of dosing (day 0).
